# Professor Stefan Hohmann (08.09.1956–02.07.2021)

**DOI:** 10.1093/femsyr/foac027

**Published:** 2022-06-30

**Authors:** Jens Nielsen, Markus J Tamás

**Affiliations:** Systems and Synthetic Biology, Chalmers University of Technology, Kemivägen 10, SE-412 96 Göteborg, Sweden. Chair of Finance and Science Committee for the International Conference on Yeast Genetics and Molecular Biology (ICYGMB); Department of Chemistry and Molecular Biology, University of Gothenburg, Box 462, SE-405 30 Göteborg, Sweden

This special issue of FEMS Yeast Research is dedicated to the memory of Professor Stefan Hohmann, one of the European pioneers of yeast molecular biology. Stefan was trained by one of the founders of yeast genetics and molecular biology, Professor Friedrich Zimmermann at the University of Darmstadt. Following his graduation, Stefan did a post doc at the Katholieke Universiteit Leuven with Professor Johan Thevelein, another world leader in the field of yeast genetics and molecular biology. Stefan continued his European journey in Gothenburg, where he influenced yeast and systems biology significantly. In Sweden, he received early on a prestigious grant from the Swedish Research Council as Research Professor, and shortly after he was appointed as Professor at the University of Gothenburg, where he played an instrumental role in gathering a number of young yeast researchers and establishing a very strong yeast research environment, impressive both by the breadth and the depth of competences. Thus today, more than 25 years after Stefan's arrival to Gothenburg, thanks to the seeds of science and leadership that he planted, there is a large and thriving research environment on yeast genetics, molecular biology, systems biology, and metabolic engineering. Similarly, Stefan also made substantial contributions to the development of yeast research in South Africa, where he spent extensive periods of time as visiting professor at the universities in Bloemfontein and Stellenbosch.

Stefan's research was focused on signal transduction events, stress responses, and transport proteins, primarily related to osmotic stress in *Saccharomyces cerevisiae*. He made many pioneering discoveries on the high osmolarity glycerol (HOG) pathway in yeast, a branched mitogen activated protein kinase (MAPK) signal transduction system that is conserved across eukaryotes. The HOG pathway is engaged in sensing osmotic stress and activating production of glycerol, which is an osmo-protectant, and with the clear in- and outputs of the pathway it has served as a model for eukaryotic MAPK pathways. Several of his research papers on the HOG pathway and its interactions stand out as influential contributions to science. Stefan also made key discoveries on transport systems involved in osmoadaptation, in particular on the role and regulation of yeast aquaporins and aquaglyceroporins. Stefan maintained a long-standing interest in yeast metabolism, and his research greatly contributed to our understanding of sugar, glycerol, and trehalose metabolism, in particular how these metabolites contribute to stress resistance. This interest led him to study other important signaling pathways and protein kinases including Snf1, which is the AMP-activated kinase of yeast. Although trained as a classical molecular biologist, Stefan was an early pioneer and supporter of systems biology. Stefan was one of the founders of the Yeast Systems Biology Network, which enabled coordination of new standards as well as leading to the foundation of several new research agendas that resulted in significant funding from both the EU and national governments in Europe.

Stefan was a great scientific mentor and he guided many young scientists in Gothenburg, Leuven, and South Africa. His international outlook and vast network of European and international scientists opened up doors and opportunities for his junior and senior colleagues. Stefan's continuous encouragement and support helped many young scientists toward scientific independence and an academic career. Many of his former students hold faculty positions in several countries carrying Stefan's legacy further.

Throughout his career, Stefan contributed to the scientific community as editor and editorial board member of several journals. He served as Editor in Chief for Current Genetics between 2001 and 2014 and Editor in Chief for Molecular Genetics and Genomics from 2005 until he passed away. Stefan chaired the International Conference on Yeast Genetics and Molecular Biology (ICYGMB) in Gothenburg both in 2003 and in 2019. With more than 1100 participants, the conference in 2003 was the conference in this series with the largest number of delegates so far, and it stands out for many in the community as a very important and memorable event. Stefan also served as chair of the Finance and Policy Committee of the International Yeast Community that is responsible for organizing ICYGMB every second year since the 1960s. Stefan was coordinator of several EC-funded research and development projects and international training projects as well as organizer of several EMBO and FEBS courses and workshops.

Besides serving his community with science, Stefan also gave his time and talent to serving as a leader. He was for several years Deputy Dean for the Natural Science Faculty at the University of Gothenburg, and in 2015 he was recruited as the Head of Department of Biology and Biological Engineering at Chalmers University of Technology. During his tenure at Chalmers the department grew, in terms of faculty, international profile, and internal strength. Stefan was a constant visionary, looking ahead on how the department could further strengthen its profile and international standing.

Stefan was much appreciated for his integrity, humor, love of his family and their animals, love for South Africa, good wines, and football. In his passion for football, Stefan showed not only the love of the game but also his caring personality, as a football coach for young boys, a ‘hobby’ that he took greatly to his heart. Stefan was a caring, engaged, dedicated, and loving person and we will all miss him tremendously, for his science, his leadership, his friendship, his humanity, his humor….

Our feelings and our thoughts go to the entire Hohmann family. We, the yeast and systems biology community of Gothenburg and of the world are here for you.

**Figure 1:**
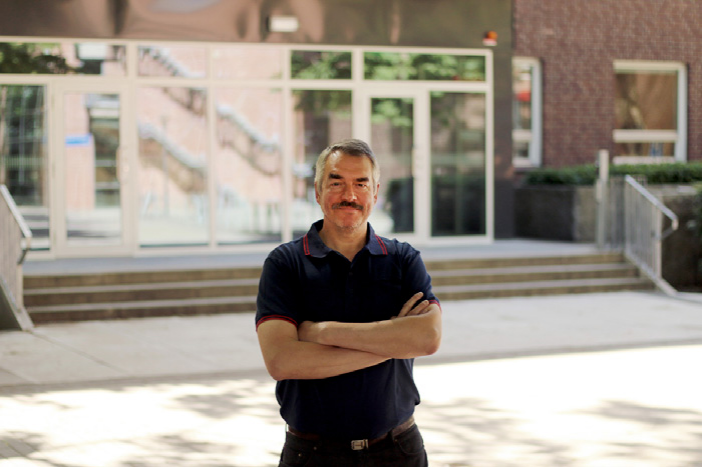
Professor Stefan Hohmann

